# Intestinal fatty acid binding protein is associated with coronary artery disease in long-term type 1 diabetes—the Dialong study

**DOI:** 10.1186/s12933-024-02509-3

**Published:** 2024-11-19

**Authors:** Marte Narum, Ingebjørg Seljeflot, Vibeke Bratseth, Tore Julsrud Berg, Kari Anne Sveen

**Affiliations:** 1 Department of Endocrinology, Morbid Obesity and Preventive Medicine, Oslo University Hospital, Oslo, Norway; 2 Institute of Clinical Medicine, Faculty of Medicine, University of Oslo, Oslo, Norway; 3 Oslo Center for Clinical Heart Research, Department of Cardiology Ullevaal, Oslo University Hospital, Oslo, Norway

**Keywords:** Type 1 diabetes, Coronary artery disease, Gut microbiota, Intestinal fatty acid binding protein, Intestinal permeability

## Abstract

**Background:**

Individuals with type 1 diabetes are at increased risk of accelerated atherosclerosis, causing coronary artery disease (CAD). The underlying mechanisms remain unclear, but new theories proposed are damage of gut mucosa causing leakage and translocation of gut microbiota products into the circulation, leading to inflammatory responses and atherosclerosis. We therefore aimed to study the associations between gut related inflammatory biomarkers and coronary atherosclerosis in individuals with long-term type 1 diabetes.

**Methods:**

In this cross-sectional, controlled study of 102 participants with type 1 diabetes and 63 control subjects, we measured circulating levels of intestinal fatty acid binding protein (I-FABP), soluble cluster of differentiation 14 (sCD14), lipopolysaccharide binding protein (LBP) and interleukin 18 (IL-18) by enzyme-linked immunosorbent assay (ELISA), and further gene expression of CD14 and toll-like receptor 4 (TLR4) by real time PCR in circulating leukocytes and peripheral blood mononuclear cells (PBMCs). The participants had either established coronary heart disease (CHD) or underwent computed tomography coronary angiography (CTCA) to assess for coronary atherosclerosis, including total, calcified and soft/mixed plaque volumes.

**Results:**

In the diabetes group, the levels of I-FABP were significantly higher in participants with established CHD or significant stenosis on CTCA compared to the participants with normal arteries or non-significant stenosis, with median 1.67 ng/ml (interquartile range [IQR] 1.02–2.32) vs. median 1.09 ng/ml (IQR 0.82–1.58), *p* = 0.003. I-FABP was associated with significant coronary artery stenosis by CTCA (> 50%) or previously established CHD in the adjusted analysis (odds ratio [OR] = 2.32, 95% confidence interval [CI]: 1.09–4.95; *p* = 0.029). The levels of I-FABP correlated also to total coronary plaque volume (*r* = 0.22, *p* < 0.05). This association remained significant after adjusting for age, sex, persistent albuminuria, eGFR, statin treatment, diabetes duration and mean time-weighted variables; HbA1c, LDL-cholesterol and systolic blood pressure (OR = 1.97, 95% CI: 1.28–3.01; *p* = 0.002).

**Conclusions:**

In this cohort of individuals with long-term type 1 diabetes I-FABP associated significantly with coronary artery stenosis, suggesting a potential role of gut mucosa damage in the process of atherosclerosis in type 1 diabetes.

**Supplementary Information:**

The online version contains supplementary material available at 10.1186/s12933-024-02509-3.

## Introduction

Individuals with type 1 diabetes have an accelerated progression of coronary atherosclerosis compared to people without diabetes, with an increased risk of developing cardiovascular disease, including coronary artery disease (CAD) [1–3]. Moreover, the cardiovascular morbidity and mortality are increased in type 1 diabetes [4–6]. However, some people have normal coronary arteries despite living with type 1 diabetes for many decades [7].

Over the past years it has been well established that inflammation is central in the development of arterial atherosclerosis, including CAD [8]. An aspect of particular interest is the gut microbiota as a potential driver for this inflammation, in an intricate interaction with the immune system and several pathways in the glucose metabolism [9, 10]. Gut mucosa damage and translocation of gut microbiota of gram-negative bacteria to the systemic circulation, importantly endotoxins or lipopolysaccharides (LPS), has been suggested to be a trigger of inflammation and insulin resistance, thereby leading to structural changes in the arterial wall and following increased cardiovascular risk [11, 12].

LPS are components of the outer membrane surface of gram-negative bacteria, promoting inflammation mainly by signaling through the Toll-like receptor 4 (TLR4) attached on cells in the innate immune system. The signaling pathway also includes the coreceptor cluster of differentiation 14 (CD14) [13, 14] and the LPS-binding protein (LBP) [12]. Interleukin 18 (IL-18), a proinflammatory cytokine, is produced by downstream signaling in the TLR signaling pathway [15]. LBP, LPS and soluble CD14 (sCD14) have been found elevated in different conditions of cardiovascular disease or populations at high risk of developing cardiovascular events [12, 16]. Previous studies have also shown LPS and other known inflammatory markers to be associated with development and progression of diabetic nephropathy in patients with type 1 diabetes, as well as components of the metabolic syndrome, including dyslipidemia and insulin resistance [17–19]. Elevated levels of IL-18 have been reported in individuals with type 1 diabetes. IL-18 has also been suggested to be involved in the development of vascular complications in diabetes [20], and as a predictor of cardiovascular events in the presence of elevated fasting glucose and in subjects with the metabolic syndrome [21].

A potential biomarker which reflects intestinal permeability and gut mucosa damage is intestinal fatty acid binding protein (I-FABP) [22, 23]. This is a cytosolic protein located in the enterocytes of the villi in the small intestine [24], which has been found to be increased in coeliac disease and necrotizing enterocolitis [25, 26]. I-FABP is pivotal in transporting fatty acids from the intestinal lumen into the enterocytes and further into the different organelles, hereby playing an important role in the lipid metabolism by maintaining a steady state of fatty acids in the intestinal epithelium. Increased permeability in the epithelium leads to release of I-FABP into the systemic circulation, making the protein a potential biomarker of enterocytic damage [27]. I-FABP has been found elevated in children and adolescents with type 1 diabetes and diabetic nephropathy [28] and was recently linked to gut dysbiosis and cardiac function in chronic heart failure [29]. However, to our knowledge, an association between I-FABP and CAD in type 1 diabetes have not been reported.

In the current study, we aimed to investigate possible associations between gut related inflammatory biomarkers including LBP, CD14, TLR4, I-FABP and IL-18 and the presence of CAD and with established CHD, in patients with long-term type 1 diabetes compared to a control group.

## Research design and methods

### Study design, participants and procedures

The Dialong study is a cross-sectional, controlled study on long-term survivors of type 1 diabetes conducted in 2015. The primary endpoints of the study are complications of long-term type 1 diabetes, including CAD and musculoskeletal complications. The inclusion criteria have previously been set out in full [30]. All patients with type 1 diabetes from 1970 or earlier attending a state funded specialized type 1 diabetes clinic, the Norwegian Diabetes Center (NDS) in Oslo, Norway, were invited. Type 1 diabetes was defined as HbA1c > 6.5% (48 mmol/mol) and evidence of lack of insulin production with fasting c-peptide concentration < 0.2 pmol/ml [30]. Out of 136 eligible patients, 103 joined the coronary artery substudy [7]. The control group (*n* = 63) without diabetes consisted of spouses/friends of the participants with type 1 diabetes. Exclusion criteria were first degree relatives and HbA1c > 6.5% (48 mmol/mol) or a known diagnosis of diabetes [30]. In the control group, three participants had previously CHD/CAD and 60 accepted to have a CTCA performed [7]. The study was approved by the regional ethics committee (reference no. 2014/851) and conformed to the Declaration of Helsinki. All participants signed an informed consent.

Background data including measurements of HbA1c, blood pressure and LDL-cholesterol values from 1980 to 2015 were collected from patient charts at NDS, clinical examination and interviews [30]. The patients had fasting blood tests, urine samples and retinal photos. The presence and degree of coronary artery disease was initially evaluated by clinical history and coronary artery calcium (CAC) imaging. The participants without previously established CHD (*n* = 88/103 in the diabetes group and *n* = 60/63 of controls) were referred to CTCA [7].

### Outcomes

All participants without previously CHD were referred to CTCA. CTCA was performed on a Dual Source CT scanner (Somatom Definition Flash, Siemens, Erlangen, Germany). The details for the CTCA investigation protocol and the definition of previous CHD have been described previously [7]. Normal coronary arteries were defined as no detected plaque in any of the coronary arteries, non-obstructive CAD (non-significant stenosis) was defined as the presence of 1–50% diameter stenosis in any of the coronary arteries, and obstructive CAD (significant stenosis) was defined as > 50% stenosis in any of the coronary arteries. The total volume of all plaques was calculated and categorized based on plaque morphology into different categories; total plaque volume, calcified plaque volume and soft/mixed plaque volume. The percentage of calcifications present determined the plaque morphology, where plaques containing more than 90% calcifications were categorized as calcified plaques and plaques containing 0–90% were categorized as soft/mixed plaques (density > 130 Hounsfield units). CAC levels were given in Agatson units [31].

### Analysis of serum sCD14, LBP and IL-18, and plasma I-FABP

Blood samples were drawn after an overnight fast. Citrated blood was kept stored on ice until it was separated by centrifugation within 30 min at 2500 g at 4 °C for 20 min. Serum was centrifugated within 1 h at 2000 g for 10 min at room temperature. I-FABP was measured in citrated plasma by ELISA (Hycult Biotech, Uden, the Netherlands). sCD14, IL-18 and LBP were measured in serum by ELISAs (R&D Systems Europe, Biotechne, Oxon, UK and Hycult Biotech). The inter-assay coefficients of variation (CV) were 3.1%, 8.0%, 8.6% and 5.5% respectively.

### Gene expression analysis of TLR4 and CD14 in circulating leukocytes and mononuclear cells

Total RNA was isolated from the PAXgene tubes with the use of PAXgene^®^ Blood RNA Kit (PreAnalytix, Qiagen, GmBH, Hilden, Germany) for gene expression of TLR4 and CD14 in circulating leukocytes. This also included an extra step of cleaning (RNeasy^®^MinElute^®^ Cleanup Kit, Qiagen). The NanoDropTM 1000 Spectrophotometer (Thermo Scientific, Wilmington, Delaware, USA) was used to examine the quality and quantity (ng/µL) of RNA. Copy DNA (cDNA) was generated by combining equal amounts of qScriptTM cDNA superMix (Quanta Biosciences, Gaithersburg, Maryland, USA) and RNA (5 ng/µl). Real time PCR was used to perform gene expression on a ViiATM7 instrument (Applied Bio-systems, Foster City, CA, USA), using TaqMan^®^ Universal PCR Master Mix (P/N 4324018) with commercially available TaqMan^®^ assay: CD14 (Hs02621496_s1) and TLR4 (Hs00152939_m1) (Applied Bio-systems). The ∆∆CT method was used to decide the mRNA levels, where relative quantification (RQ) was given by using β2-microglobulin (HS99999907_m1) (Applied Biosystems) as an endogenous control [32]. This method of measuring the gene expression of TLR4 has also been reported previously [33].

PBMCs were collected from a sub-set of the participants, randomly included (*n* = 18 in the diabetes group and *n* = 14 in the control group), by use of Cell Preparation Tube (CPT) with sodium citrate (Becton, Dickinson and Company, New Jersey, US). These were isolated within 2 h by centrifugation at several steps in room temperature. First, once at 1500 g for 20 min, and thereafter the mononuclear layer was pipetted off and centrifuged twice at 300 g for 10 min at room temperature. Any remaining plasma was pipetted off before freezing of the PBMCs pellets at − 80 °C until analyses. The gene expression analysis of TLR4 and CD14 in the PBMCs was performed as described for circulating leukocytes.

### Mean time-weighted variables– HbA1c, LDL-c and systolic blood pressure

Longitudinal HbA1/HbA1c values were available from 1980 to 2015, and the calculations of mean time-weighted HbA1c and “Estimated Full Duration HbA1c” (EFD HbA1c) have been described previously [30]. The calculations of mean time-weighted LDL-c and systolic blood pressure up until 2015 have also been described previously [7].

### Statistical analysis

The initial power analysis for the Dialong study was based on skin collagen levels of the AGE product glucosepane and its association with CAD. It suggested a need of 77 participants with diabetes to detect a significant difference in glucosepane levels between patients with obstructive CAD and patients with normal coronary arteries (power 90%, probability 0.05). This was based on a distribution in diabetes patients of 30%, 40% and 30% with absent CAD, non-obstructive CAD and obstructive CAD respectively, in addition to a previous analysis from the Oslo study which showed mean glucosepane levels to be 500 pmol/L higher in the group with obstructive CAD versus absent CAD [34, 35]. This power analysis was based only on participants with obstructive disease and normal coronary arteries. To avoid loss of power in the current study, it was decided to keep all patients with non-obstructive CAD (60%). Cases with missing data were excluded.

Continuous variables and their distribution were assessed by histograms and Q-Q plots and were presented as mean (standard deviation) or median (interquartile range). Skewed variables were natural log (ln) transformed before statistical analysis. Categorical variables were presented as number of individuals (percentages). Clinical characteristics, levels of sCD14, LBP, IL-18, TLR4 and I-FABP and outcomes on CTCA were compared between the groups using independent two-tailed Student’s t-test or Mann-Whitney U test for continuous data, for symmetrically distributed data and non-normally distributed data, respectively. χ^2^ was used for categorical data. Differences between more than two groups were analyzed by ANOVA for normal distributed data and by Kruskal-Wallis test for non-normally distributed data. Spearman correlation analyses were performed to assess correlations between the biomarkers and the plaque volume measures on CTCA or the CAC score. Linear regression analyses were performed to adjust for confounders. Dependent variables without normally distributed residuals in linear regression analyses were natural log (ln) transformed, and the coefficients of the independent variables were then exponentiated. Binary logistic regression analyses were used to calculate odds ratios (ORs), and both univariate and multivariate analyses were performed to adjust for possible confounders. Variables for the model were chosen based on significant associations in the univariable analyses with the biomarkers or previously knowledge on associations with the outcome measurements. Age, sex, persistent albuminuria, and mean time-weighted variables HbA1c, LDL-cholesterol and systolic blood pressure were the variables found most suitable for the model. P values ≤ 0.05 were considered statistically significant. All statistical analyses were performed using SPSS Statistics version 29 (IBM Corp., Armonk, NY).

## Results

Demographical and clinical characteristics in the diabetes group and the control group are shown in Table 1. The group with diabetes had significantly higher systolic blood pressure and lower LDL-cholesterol levels compared to the control group, as previously reported [7]. One participant in the diabetes group was excluded from the analysis due to missing data on the gut related inflammatory biomarkers, leaving 102 participants in the diabetes group and 63 controls included in the final analysis.

**Table 1:** Clinical characteristics of the study population, the Dialong study

	Diabetes subjects with obstructive CAD (*n* = 21) or previously established CHD (*n* = 15)	Diabetes subjects without obstructive CAD or CHD (*n* = 66)	Controls (*n* = 63)	*P* value	
**Demographics**				a	b
Age	64.0 ± 7.0	61.0 ± 6.9	62.7 ± 7.0	**0.036**	0.59
Sex, male	21 (58.3%)	30 (45.5%)	28 (44.4%)	0.30^z^	0.52
Daily smoker	2 (5.6%)	2 (3.0%)	6 (9.5%)	0.61^z^	0.18
LDL-c, mmol/L	2.6 ± 0.8	2.8 ± 0.8	3.8 ± 1.0	0.27	< **0.001**
Systolic blood pressure, mmHg	146 ± 21	145 ± 19	138 ± 20	0.89	**0.012**
Diastolic blood pressure, mmHg	74 ± 9	75 ± 8	81 ± 10	0.38	< **0.001**
Body mass index, kg/m^2^, median (IQR)	25.9 (23.5–30.4)	25.6 (23.3–27.8)	24.8 (22.6–27.7)	0.43	0.34
eGFR^x^	82 ± 21	86 ± 19	82 ± 14	0.32	0.33
Triglycerides, mmol/L, median (IQR)	0.8 (0.6-1.0)	0.8 (0.6-1.0)	0.9 (0.7–1.3)* (*62)	0.47	**0.003**
NT-proBNP, ng/L	109 (43–212) *(*n* = 21)	52 (28–126)	59 (35–82)* (*59)	0.094	0.17
Statin treatment	27 (75.0%)	27 (40.9%)		**0.002**	**< 0.001**
ACE/ARB-inhibitor	26 (72.2%)	24 (36.4%)	9 (14.3%)	**< 0.001**	**< 0.001**
Coeliac disease	3 (8.3%)	3 (4.5%)	13 (20.6%)	0.66	**0.015**
Spot urinary ACR	10 (30.3%)* (*33)	8 (12.1%)	3 (4.8%)	0.05	
**Diabetes related factors**					
Diabetes duration, years median (IQR)	52 (47–56)	48 (46–52)		**0.018**	
HbA1c, estimated full duration, %, mmol/mol	8.3 ± 0.7, 67 ± 7.5	7.8 ± 0.8, 62 ± 8.5		**0.004**	
HbA1c, mean time-weighted, %, mmol/mol	8.1 ± 0.7, 65 ± 8	7.7 ± 0.7, 61 ± 7.5		**0.016**	
Current HbA1c (2015), %, mmol/mol	7.6 ± 0.9, 60 ± 9.5	7.3 ± 0.7, 56 ± 7.5	5.5 ± 0.3* (*61) 37 ± 3.5	**0.036**	**< 0.001**
Mean time-weighted LDL-c, mmol/L	3.0 ± 0.7	2.8 ± 0.6		0.34	
Mean time-weighted systolic blood pressure, mmHg	133 ± 11	129 ± 10		0.065	
Retinopathy, none	1 (2.8%)	4 (6.1%)		0.65^z^	
Background	15 (41.7%)	37 (56.1%)			
Proliferative	20 (55.6%)	25 (37.9%)			
Persistent albuminuria	11 (30.6%)	7 (10.6%)		**0.016** ^**z**^	
Clinical neuropathy (likely)	29 (80.6%)	39 (59.1%)		0.059	

Data are presented as mean ± SD, n (%), median (IQR). ^z^Fischer’s Exact Test. ^x^estimated glomerular filtration rate calculated by MDRD formula. a = P values indicate differences between diabetes subjects with obstructive CAD or previously established CHD, versus diabetes subjects with normal coronary arteries or nonsignificant CAD. b = P values indicate differences between the total diabetes group versus the control group. * = n

### Levels of I-FABP, gut related inflammatory biomarkers and coronary artery stenosis or established CHD in the diabetes group

Levels of gut related inflammatory biomarkers in the diabetes group with established CHD or significant stenosis (> 50%) on CTCA compared to those with normal arteries or non-significant stenosis (1–50%), are presented in Table 2. The levels of I-FABP were significantly higher in the participants with established CHD or significant stenosis (> 50%) compared to those with normal coronary arteries or nonsignificant stenosis (< 50%), median 1.67 ng/ml (IQR 1.02–2.32) versus 1.09 ng/ml (IQR 0.82–1.58), (*p* = 0.003). In the small sub-set of the participants with isolated PBMCs available, we also found a significant difference in the gene expression levels of CD14, with significantly higher levels in the participants with established CHD or significant stenosis on CTCA compared to the participants with normal arteries or non-significant stenosis, median 3.8 RQ (IQR 3.1–5.9) versus median 0.9 RQ (IQR 0.6–1.4).

**Table 2:** Gut related inflammatory biomarkers in the total study population, the Dialong study. * = n

	Diabetes subjects with obstructive CAD or CHD (*n* = 36)	Diabetes subjects without obstructive CAD or CHD (*n* = 66)	Controls (*n* = 63)	*P* value	
Inflammatory circulating biomarkers				a	b
CD14, ng/ml	1461 (1315–1673)	1383 (1241–1657)	1294 (1132–1443)	0.21	< **0.001**
LBP, ng/ml	10,677 (8750–12644)	10,260 (8178–11893)	10,245 (8891–12695)	0.28	0.99
I-FABP, ng/ml	1.67 (1.02–2.32)	1.09 (0.82–1.58)	0.82 (0.67–1.08)* (*62)	**0.003**	< **0.001**
IL-18, pg/ml	255 (210–352) *(*n* = 35)	248 (184–318)	248 (188–312)	0.30	0.75
Gene expression in circulating leukocytes					
CD14_CL, RQ	0.9 (0.7–1.2)* (*35)	1.0 (0.7–1.3)* (*63)	0.9 (0.7–1.3)* (*59)	0.32	0.51
TLR4_CL, RQ	0.9 (0.8–1.3)* (*35)	1.0 (0.7–1.3)* (*63)	0.9 (0.7–1.3)* (*59)	0.89	0.97
Gene expression in PBMCs					
CD14_PBMC, RQ	3.8 (3.1–5.9)* (*4)	0.9 (0.6–1.4)* (*14)	1.4 (1.0-2.3)* (*14)	**0.005**	0.54
TLR4_PBMC, RQ	1.6 (1.0–2.0)* (*4)	1.1 (0.8–1.3)* (*14)	1.3 (1.0-1.5)* (*14)	0.13	0.44

Data are presented as median (IQR). RQ: Relative Quantification. a = P values indicate differences between diabetes subjects with obstructive CAD or previously established CHD, versus diabetes subjects with normal coronary arteries or nonsignificant CAD. b = P values indicate differences between the total diabetes group versus the control group

**Fig. 1:**
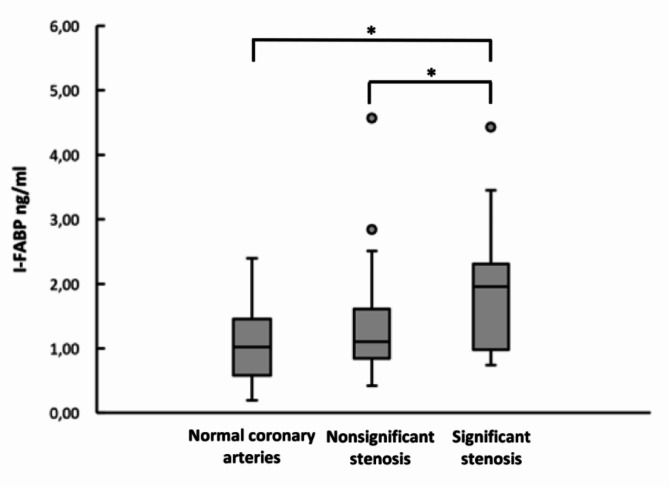
Levels of I-FABP in type 1 diabetes with significant stenosis (*n* = 21), nonsignificant stenosis (*n* = 52) and normal coronary arteries (*n* = 14) on CTCA. The values are presented as median (IQR). I-FABP levels are given in ng/ml. *P < 0.05 (Kruskal-Wallis test). ● = outliers

In further subgroup analysis of individuals with diabetes without established CHD, performing CTCA (*n* = 87), the levels of I-FABP in those with significant stenosis were higher compared to those with normal coronary arteries, median 1.96 ng/ml (IQR 0.98–2.31) versus 1.02 ng/ml (IQR 0.58–1.46), *p* = 0.023. The levels of I-FABP were also found higher in the group with significant stenosis compared to the intermediate group with nonsignificant stenosis, median 1.11 ng/ml (IQR 0.84–1.61), (*p* = 0.024) (Fig. 1). There were no significant findings comparing sCD14, LBP, IL-18 and gene expression of CD14 and TLR4 in circulating leukocytes. Due to collection of circulating PBMCs only in a sub-set of the participants, there were not enough participants within the diabetes CTCA subgroup to analyze for gene expression of CD14 and TLR4 in circulating PBMCs.

### Correlations between I-FABP and cardiovascular risk factors

In the diabetes group, I-FABP was negatively correlated with eGFR (*r* = -0.336, *p* < 0.01) and with LDL-cholesterol (*r* = -0.207, *p* < 0.05). I-FABP did not correlate significantly with systolic blood pressure, cross-sectional HbA1c or mean time-weighted HbA1c, neither with statin treatment nor diabetes duration. There were no significant correlations between I-FABP and cardiovascular risk factors in the control group (Supplementary Table 3).

### Associations of I-FABP with measures of coronary artery stenosis in the diabetes group

The levels of I-FABP were correlated to coronary total plaque volume (*r* = 0.22, *p* < 0.05) in the diabetes group, as well as with CAC score (*r* = 0.24, *p* < 0.05) and total mixed/soft plaque volume (*r* = 0.27, *p* < 0.05) on CTCA imaging. The association with total plaque volume remained significant after adjustments of age, sex, persistent albuminuria, eGFR, statin treatment, diabetes duration and mean time-weighted variables; HbA1c, LDL-cholesterol and systolic blood pressure (adjusted r^2^ = 0.524 in linear regression analysis) (Table 3). The associations with CAC score also remained significant after adjusting for the same variables (r^2^ = 0.496), (Supplementary Table 1). Other gut related inflammatory biomarkers were not found to associate with plaque volume measures or CAC score.

**Table 3:** Simple and multiple regression models for the association between I-FABP and total plaque volume in the diabetes group. r^2^ = 0.524. *N* = 85

Total Plaque Volume (ln)	B	Unadjusted 95% CI	*P* value	B	Adjusted 95% CI	*P* value
I-FABP	1.96	(1.20–3.20)	0.008	1.97	(1.28–3.01)	0.002
Age	1.10	(1.04–1.16)	< 0.001	1.08	(1.02–1.14)	0.008
Sex	3.72	(1.72–8.06)	0.001	2.89	(1.50–5.57)	0.002
Persistent albuminuria	3.34	(1.14–9.79)	0.028	2.25	(0.95–5.34)	0.065
LDL-c, mean time-weighted	2.71	(1.42–5.18)	0.003	1.90	(1.11–3.24)	0.019
HbA1c, mean time-weighted	1.38	(0.80–2.36)	0.24	1.14	(0.73–1.78)	0.568
Systolic blood pressure, mean time-weighted	1.07	(1.03–1.11)	0.001	1.01	(0.98–1.05)	0.422
eGFR	1.00	(0.98–1.02)	0.919	1.01	(1.00-1.03)	0.129
Statin treatment	2.50	(1.12–5.55)	0.025	1.27	(0.66–2.44)	0.472
Diabetes duration	1.18	(1.09–1.29)	< 0.001	1.09	(1.00-1.19)	0.030

**Fig. 2:**
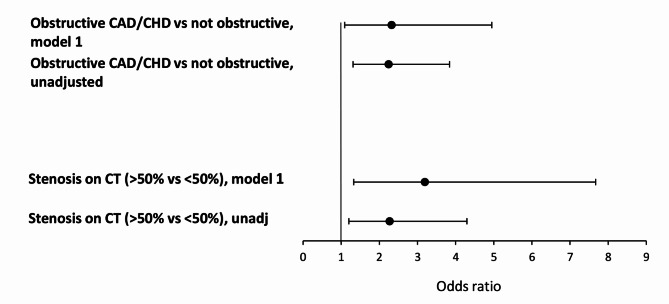
Odds ratios (OR) and 95% confidence intervals of I-FABP on significant stenosis (> 50%) on CTCA or obstructive CAD/established CHD in the diabetes group, unadjusted and adjusted models. Model 1: Adjusted for age, sex, persistent albuminuria, eGFR, statin treatment, diabetes duration, LDL-c mean time-weighted, HbA_1c_ mean time-weighted and systolic blood pressure mean time-weighted

In the diabetes group, I-FABP associated with the presence of obstructive CAD or previously established CHD unadjusted (odds ratio unadjusted [OR_unadj_] 2.25, 95% CI: 1.31–3.84, *p* = 0.003). The association remained significant after adjusting for relevant covariates, (OR adjusted [OR_adj_] 2.32, 95% CI: 1.09–4.95, *p* = 0.029). I-FABP also associated with the presence of > 50% coronary artery stenosis on CTCA (vs. < 50% stenosis) unadjusted (OR unadjusted [OR_unadj_] 2.27), 95% CI: 1.20–4.30, *p* = 0.012). This association was even stronger in the adjusted analysis, (OR adjusted [OR_adj_] 3.19, 95% CI: 1.33–7.67, *p* = 0.009) (Fig. 2, Supplementary Table 2).

### Levels of gut related inflammatory biomarkers in the control group

There were significantly higher levels of I-FABP and sCD14 in the diabetes group compared to the control group, respectively (*p* < 0.001, both) (Table 2). There were no significant differences in the levels of LBP or IL-18. Also, no significant differences were found in gene expression of CD14 and TLR4 in circulating leukocytes and PBMCs between the diabetes group and the control group (Table 2). In the control group, there were no significant differences in levels of I-FABP in the subjects with significant stenosis versus subjects with normal coronary arteries on CTCA, and also no significant correlations between I-FABP and plaque volume measures or CAC score.

## Discussion

In the present study, we found significantly higher plasma levels of I-FABP in long-term type 1 diabetes individuals with obstructive CAD and with previously established CHD compared to individuals with nonobstructive disease or normal coronary arteries. The levels of I-FABP were also found significantly higher in the individuals with long-term type 1 diabetes compared to controls. In the diabetes group, I-FABP also associated with significant coronary artery stenosis by CTCA with an about 2.7-fold higher risk for having coronary stenosis with high levels of I-FABP, as well as with measure of total plaque volume of the coronary arteries. This association remained significant after adjusting for relevant covariates, including mean time-weighted HbA1c and statin treatment. Rosuvastatin has previously been found to decrease levels of I-FABP [36], and treatment of statins is clearly linked to the risk of CAD. Still, the association of I-FABP with coronary atherosclerosis remained significant after adjusting for statin treatment in the current study.

The reason for a lack of an association with mean time-weighted HbA1c and coronary atherosclerosis as earlier shown [3, 5] is unknown. One reason could be that the Dialong participants are a cohort of long-term survivors of type 1 diabetes with less hyperglycemia compared to a whole cohort including non-survivors of hyperglycemia. I-FABP was neither found to associate with HbA1c levels, which also possibly could be explained by the relatively small sample size. Further, the individuals in the present cohort have a near optimal glycemic level in terms of HbA1c.

These findings suggest that damage of gut mucosa may play a role in the formation of atherosclerosis in type 1 diabetes. Generally, alterations in the composition of the gut microbiota leading to enterocytic damage and thereby leaky gut have been proposed to be a contributive factor to the development of inflammation and furthermore CAD [37, 38]. However, the relationship between chronic hyperglycemia and intestinal barrier impairment is debated [39].

Increasing evidence points towards a pivotal role of the gut microbiota in the development of CAD, suggesting gut microbial changes to reduce the production of butyrate, which may lead to further alterations in the inflammatory pathways [40]. Gut mucosa barrier dysfunction may also promote leakage of microbial toxins including LPS. Several studies have shown LPS, LBP and sCD14 to be elevated in individuals with cardiovascular disease or populations at high risk of developing cardiovascular events [12, 16, 33, 41]. However, only a relatively small number of studies have investigated the impact of gut leakage and metabolic endotoxemia in type 1 diabetes. Some of these studies have primarily been focusing on the metabolic endotoxemia and the end products of this, in general LPS and the related substances in the LPS-TLR4 pathway [17, 42]. A study on an Indian population found increased serum levels of LPS, but decreased levels of LBP in a type 1 diabetes group compared to controls with normal glucose tolerance. In the same study no significant difference was found in the levels of these markers between the subjects with and without macrovascular disease within the diabetes group [43]. Notably, the diagnosis of CAD in this study was based on previous history of myocardial infarction combined with drug treatment for CAD or electrocardiographic changes suggesting CAD. Thus, it could be argued that the precision of the diagnostic tool according to discover undiagnosed CAD was not optimal. Still, the evidence regarding the impact of metabolic endotoxemia on cardiovascular disease is conflicting [37, 38]. Interestingly, the findings in our present study did not reveal significant differences in LBP, sCD14 or related substances in the TLR pathway between type 1 diabetes individuals with and without CAD or established CHD. This contrasts with previous studies suggesting a role of inflammation on CAD in general and in type 1 diabetes. The lack of significance in the above-mentioned biomarkers in the current study could be explained by a type II error due to the relatively small sample size.

Only a few studies have investigated the link between I-FABP and CAD in general. In a Norwegian randomized controlled study on patients with suspected CAD, no significant differences in the levels of I-FABP were found, neither at baseline nor after performing strenuous exercise [33]. In the present study, we demonstrated elevated levels of I-FABP in individuals with type 1 diabetes compared to controls. Additionally, I-FABP associated with significant coronary artery stenosis by CTCA and with total plaque volume. Within the diabetes group, the higher levels found among the individuals with significant CAD or established CHD compared to the individuals with nonsignificant CAD or normal coronary arteries, support a link between I-FABP and type 1 diabetes in general, but also with CAD.

In a cohort of pregnant women with type 1 diabetes, levels of I-FABP were elevated, in parallel with changes in the gut microbiome during pregnancy [44]. I-FABP was also associated with diabetes nephropathy in a population of children and adolescents with type 1 diabetes. In this cohort, I-FABP also associated significantly with carotid intima media thickness, also among the subjects without diabetic nephropathy [28]. Thus, diabetes nephropathy could not fully explain the findings of subclinical atherosclerosis among the individuals in this cohort. This is in line with our findings, where persistent albuminuria and/or decreased eGFR did not mask the significance of I-FABP in the presence of CAD. This suggests a role of gut mucosa damage in the process of atherosclerosis in type 1 diabetes, separately from the presence of diabetes nephropathy.

Some studies have shown I-FABP to be elevated in conditions reflecting acute intestinal injury, including acute mesenteric ischemia [45], necrotizing enterocolitis [26] and strangulated small bowel obstruction [46]. The relationship and coexistence between type 1 diabetes and coeliac disease is well-known [47, 48]. I-FABP levels were found higher in a Polish pediatric type 1 diabetes cohort compared to the controls, and the levels were also comparable in the subgroups with both active coeliac disease and type 1 diabetes, and those diagnosed with type 1 diabetes only. This indicates disruption of the gut mucosa epithelium in type 1 diabetes to be independent of the presence of coeliac disease [49]. Thus, it could be hypothesized that this disruption could lead to chronic low-grade inflammation, applicable to the development of atherosclerosis and furthermore potential CAD. However, the heart-gut crosstalk is an evolving field of study with complex interactions between different factors, including gut microbiota, diet and inflammatory markers. Previous research has primarily focused on the causal pathway of gut dysbiosis effect on cardiovascular disease [50]. On the other side, the potential of reverse causation should be addressed. An atherosclerotic proinflammatory environment could possibly affect the leukocytes in the intestinal wall and further influence gut microbiota inflammation. Therefore, due to the cross-sectional design, the observed augmentation in levels of I-FABP in individuals with obstructive CAD and previously established CHD in the current study, could also hypothetically be explained by a potential reverse causation.

This study has some limitations. Firstly, the study has a cross-sectional design, which makes it not applicable to draw conclusions of causality. Secondly, we did not measure LPS per se which could have given more insight into the toxin leakage theory, and measures of the gut microbiome could possibly have confirmed some of the suggested links in this study. Another connected limitation is the missing information on dietary habits. Also, PBMCs were obtained from only 18 individuals with type 1 diabetes and 14 controls. Thirdly, there are supplemental methods that potentially could be more specific in measuring gut inflammation than the methods used in this study. Fourthly, the sample size of the study, which is relatively small and increases the risk of false-negative findings. Still, the association of I-FABP with type 1 diabetes in general is in line with other previous studies investigating I-FABP in cohorts of type 1 diabetes. The strengths of the current study are the detailed measurements of coronary atherosclerosis and long-time hyperglycemia in a unique study population with long-term type 1 diabetes including a control group.

## Conclusions

In conclusion, we demonstrate a significantly higher risk of having CAD or established CHD with high levels of I-FABP in individuals with long-term type 1 diabetes. These results propose a potential role of gut mucosa damaged in the process of atherosclerosis in type 1 diabetes. Future research on gut involvement in the pathophysiology of CAD in type 1 diabetes is needed to support these hypotheses.

## Electronic supplementary material

Below is the link to the electronic supplementary material.

Supplementary Material 1

## Data Availability

The datasets used and analyzed during the current study are available from the corresponding author on reasonable request.

## Abbreviations

ACR: Albumin to creatinine ratio
AGE: Advanced glycemic end product
CPT: Cell preparation tube
CL: Circulating leukocytes
CV: Coefficient of variation
CI: Confidence interval
cDNA: Copy DNA
CAC: Coronary artery calcium
CAD: Coronary artery disease
CHD: Coronary heart disease
CTCA: Computed tomography coronary angiography
ELISA: Enzyme-linked immunosorbent assay
EFD HbA1c: Estimated full duration HbA1c
eGFR: Estimated glomerular filtration rate
HbA1c: Glycated hemoglobin
HLA: Human leukocyte antigen
IL-18: Interleukin 18
IQR: Interquartile range
I-FABP: Intestinal fatty acid binding protein
LPS: Lipopolysaccharides
LBP: Lipopolysaccharide binding protein
LDL: Low-density lipoprotein
NDS: Norwegian Diabetes Center
OR: Odds ratio
PCR: Polymerase chain reaction
PBMC: Peripheral blood mononuclear cell
RQ: Relative quantification
sCD14: Soluble cluster of differentiation 14
TLR4: Toll-like receptor 4
