# Correction: Intestinal fatty acid binding protein is associated with coronary artery disease in long-term type 1 diabetes—the Dialong study

**DOI:** 10.1186/s12933-025-02907-1

**Published:** 2025-09-23

**Authors:** Marte Narum, Ingebjørg Seljeflot, Vibeke Bratseth, Tore Julsrud Berg, Kari Anne Sveen

**Affiliations:** 1 Department of Endocrinology, Morbid Obesity and Preventive Medicine, Oslo University Hospital, Oslo, Norway; 2 Institute of Clinical Medicine, Faculty of Medicine, University of Oslo, Oslo, Norway; 3 Oslo Center for Clinical Heart Research, Department of Cardiology Ullevaal, Oslo University Hospital, Oslo, Norway

**Correction to: Cardiovascular Diabetology (2024) 23:419** 10.1186/s12933-024-02509-3

In the original publication of this article [1], the following errors occurred in Table 1 which have been listed below:

In Table 1, under statin treatment row, the corresponding numbers in Controls column is: 9 (14.3%)

In ACE/ARB-inhibitor row, the correct corresponding numbers in Controls column is: 13 (20.6%)

In coeliac disease row, the correct corresponding numbers in Controls column is: No value applicable (blank space). The correct corresponding numbers in P value under b column is: No value applicable (blank space)

Regarding spot urinary ACR, the correct corresponding numbers in P value under b column is: 0.015

The correct Table 1 is as follows:

**Table 1:** Clinical characteristics of the study population, the Dialong study

	Diabetes subjects with obstructive CAD (*n* = 21) or previously established CHD (*n* = 15)	Diabetes subjects without obstructive CAD or CHD (*n* = 66)	Controls (*n* = 63)	*P* value	
**Demographics**				a	b
Age	64.0 ± 7.0	61.0 ± 6.9	62.7 ± 7.0	**0.036**	0.59
Sex, male	21 (58.3%)	30 (45.5%)	28 (44.4%)	0.30^z^	0.52
Daily smoker	2 (5.6%)	2 (3.0%)	6 (9.5%)	0.61^z^	0.18
LDL-c, mmol/L	2.6 ± 0.8	2.8 ± 0.8	3.8 ± 1.0	0.27	< **0.001**
Systolic blood pressure, mmHg	146 ± 21	145 ± 19	138 ± 20	0.89	**0.012**
Diastolic blood pressure, mmHg	74 ± 9	75 ± 8	81 ± 10	0.38	< **0.001**
Body mass index, kg/m^2^, median (IQR)	25.9 (23.5–30.4)	25.6 (23.3–27.8)	24.8 (22.6–27.7)	0.43	0.34
eGFR^x^	82 ± 21	86 ± 19	82 ± 14	0.32	0.33
Triglycerides, mmol/L, median (IQR)	0.8 (0.6–1.0)	0.8 (0.6–1.0)	0.9 (0.7–1.3)* (*62)	0.47	**0.003**
NT-proBNP, ng/L	109 (43–212) *(n = 21)	52 (28–126)	59 (35–82)* (*59)	0.094	0.17
Statin treatment	27 (75.0%)	27 (40.9%)	9 (14.3%)	**0.002**	**< 0.001**
ACE/ARB-inhibitor	26 (72.2%)	24 (36.4%)	13 (20.6%)	**< 0.001**	**< 0.001**
Coeliac disease	3 (8.3%)	3 (4.5%)		0.66	
Spot urinary ACR	10 (30.3%)* (*33)	8 (12.1%)	3 (4.8%)	0.05	**0.015**
**Diabetes related factors**					
Diabetes duration, years median (IQR)	52 (47–56)	48 (46–52)		**0.018**	
HbA1c, estimated full duration, %, mmol/mol	8.3 ± 0.7, 67 ± 7.5	7.8 ± 0.8, 62 ± 8.5		**0.004**	
HbA1c, mean time-weighted, %, mmol/mol	8.1 ± 0.7, 65 ± 8	7.7 ± 0.7, 61 ± 7.5		**0.016**	
Current HbA1c (2015), %, mmol/mol	7.6 ± 0.9, 60 ± 9.5	7.3 ± 0.7, 56 ± 7.5	5.5 ± 0.3* (*61) 37 ± 3.5	**0.036**	**< 0.001**
Mean time-weighted LDL-c, mmol/L	3.0 ± 0.7	2.8 ± 0.6		0.34	
Mean time-weighted systolic blood pressure, mmHg	133 ± 11	129 ± 10		0.065	
Retinopathy, none	1 (2.8%)	4 (6.1%)		0.65^z^	
Background	15 (41.7%)	37 (56.1%)			
Proliferative	20 (55.6%)	25 (37.9%)			
Persistent albuminuria	11 (30.6%)	7 (10.6%)		**0.016**^**z**^	
Clinical neuropathy (likely)	29 (80.6%)	39 (59.1%)		0.059	

Data are presented as mean ± SD, n (%), median (IQR). ^z^Fischer’s Exact Test. ^x^estimated glomerular filtration rate calculated by MDRD formula. a = P values indicatedifferences between diabetes subjects with obstructive CAD or previously established CHD, versus diabetes subjects with normal coronary arteries or nonsignificantCAD. b = P values indicate differences between the total diabetes group versus the control group. * = n
