# Supplementary material for: Intestinal fatty acid binding protein is associated with coronary artery disease in long-term type 1 diabetes—the Dialong study
Source: Cardiovasc Diabetol. 2024 Nov 19;23:419. doi: 10.1186/s12933-024-02509-3 (PMC11575117; doi:10.1186/s12933-024-02509-3)
Supplement: Supplementary file 1 — Supplementary Material 1 [file 12933_2024_2509_MOESM1_ESM.pdf]

Supplementary table 1: Simple and multiple regression models for the association between I-FABP and CAC score in the diabetes group. $r^2 = 0.496$. n = 86.

CAC score (ln)	B	Unadjusted 95 % CI	P value	B	Adjusted 95 % CI	P value
I-FABP	2.50	(1.31-4.76)	0.006	2.33	(1.30-4.15)	0.005
Age	1.16	(1.08-1.24)	< 0.001	1.13	(1.05-1.21)	0.001
Sex	3.65	(1.28-10.40)	0.016	3.23	(1.34-7.81)	0.010
Persistent albuminuria	3.82	(0.91-16.06)	0.067	3.03	(0.93-9.81)	0.065
LDL-c, mean time-weighted	2.96	(1.24-7.08)	0.015	1.95	(0.94-4.02)	0.072
HbA_{1c}, mean time-weighted	1.17	(0.57-2.40)	0.659	0.96	(0.52-1.76)	0.891
Systolic blood pressure, mean time-weighted	1.09	(1.03-1.14)	0.001	1.01	(0.97-1.06)	0.632
eGFR	0.99	(0.96-1-02)	0.473	1.01	(0.98-1.03)	0.649
Statin treatment	5.76	(2.08-15.94)	< 0.001	2.74	(1.13-6.69)	0.027
Diabetes duration	1.24	(1.11-1.39)	< 0.001	1.08	(0.97-1.20)	0.181

Supplementary table 2: Odds ratios (OR) and 95% confidence interval (CI) of significant stenosis on CT (>50%) or obstructive CAD/CHD, unadjusted and adjusted models, in the diabetes group.
Model 1: Adjusted for age, sex, persistent albuminuria, LDL-c mean time-weighted, HbA1c mean time-weighted, systolic blood pressure mean time-weighted, eGFR, statin treatment and diabetes duration.

		**I-FABP ng/ml**		**P value**
Significant stenosis on CT / nonsignificant stenosis on CT (n = 87)	Unadj	OR (95 % CI)	2.27 (1.20-4.30)	0.012
	Model 1	OR (95 % CI)	3.19 (1.33-7.67)	0.009
Obstructive CAD/CHD / not obstructive CAD/CHD (n = 102)	Unadj	OR (95 % CI)	2.25 (1.31-3.84)	0.003
	Model 1	OR (95 % CI)	2.32 (1.09-4.95)	0.029

Supplementary table 3: Coefficients of correlations (Spearman's rho) of I-FABP with cardiovascular risk factors. *Significant at the 0.05 level. **Significant at the 0.01 level.

	I-FABP (ng/ml)	
	Type 1 diabetes r-value	Controls r-value
**Age**	0.038	-0.071
**HbA1c**	-0.113	0.049
**LDL-c**	-0.207*	0.049
**Systolic blood pressure**	0.027	-0.075
**Diastolic blood pressure**	0.048	-0.13
**eGFR**	-0.336**	-0.009
**Triglycerides**	-0.124	0.080
**NT-proBNP**	0.114	-0.239
**BMI**	-0.004	0.069
**Statin treatment**	0.175	-0.020
**LDL-c, mean time-weighted**	-0.056	
**HbA1c, mean time-weighted**	-0.068	
**Systolic blood pressure, mean time-weighted**	0.136	
**Diabetes duration**	0.152	

Supplementary table 4: Odds ratios (OR) and 95% confidence interval (CI) of significant stenosis on CT (>50%) or obstructive CAD/CHD, unadjusted and adjusted models, in the whole study population, the diabetes group and control group combined.
Model 1: Adjusted for diabetes/group, age, sex, eGFR and statin treatment.

		**I-FABP ng/ml**		**P value**
Significant stenosis on CT / nonsignificant stenosis on CT (n = 146)	Unadj	OR (95 % CI)	2.15 (1.26-3.67)	0.005
	Model 1	OR (95 % CI)	1.95 (1.04-3.67)	0.038
Obstructive CAD/CHD / not obstructive CAD/CHD (n = 165)	Unadj	OR (95 % CI)	2.20 (1.40-3.45)	< 0.001
	Model 1	OR (95 % CI)	1.84 (1.05-3.22)	0.033

Supplementary table 5: Simple and multiple regression models for the association between I-FABP and Total Plaque Volume in the whole study population, the diabetes group and control group combined.
$r^2 = 0.474$. n = 144.

Total Plaque Volume (ln)	B	Unadjusted 95 % CI	P value	B	Adjusted 95 % CI	P value
I-FABP	2.33	(1.51-3.57)	<0.001	1.64	(1.13-2.39)	0.010
Diabetes	6.62	(3.66-11.94)	<0.001	4.48	(2.57-7.83)	<0.001
Age	1.07	(1.03-1.13)	0.002	1.08	(1.04-1.12)	<0.001
Sex	4.43	(2.40-8.19)	<0.001	3.55	(2.12-5.92)	<0.001
eGFR	1.01	(0.99-1.03)	0.236	1.00	(0.99-1.02)	0.579
Statin treatment	5.08	(2.63-9.81)	<0.001	1.88	(1.04-3.40)	0.035
